# Supplementary material for: Factors influencing inappropriate use of antibiotics: Findings from a nationwide survey of the general public in Malaysia
Source: PLoS One. 2021 Oct 20;16(10):e0258698. doi: 10.1371/journal.pone.0258698 (PMC8528291; doi:10.1371/journal.pone.0258698)
Supplement: S1 File — (PDF) [file pone.0258698.s001.pdf]

KAJIAN MENGENAI PENGETAHUAN, SIKAP, DAN AMALAN TERHADAP PENGGUNAAN DAN KETAHANAN ANTIBIOTIK DI KALANGAN ORANG AWAM

A SURVEY ON KNOWLEDGE, ATTITUDES AND PRACTICES TOWARDS ANTIBIOTIC USE AND RESISTANCE AMONG THE GENERAL PUBLIC

Bahagian A/ Section A<br><br>MAKLUMAT AM/ GENERAL INFORMATION		
A1	Umur/ Age	____________ tahun/ years old
A2	Jantina/ Gender	[1] Lelaki/ Male<br>[2] Perempuan/ Female
A3	Bangsa/ Ethnicity	[1] Melayu/ Malay<br>[2] Cina/ Chinese<br>[3] India/ Indian<br>[4] Lain-lain, sila nyatakan jawapan di soalan A.3_o/ Others, please specify the answer in A.3_o
A3_o	Lain-lain bangsa, sila nyatakan/ Other ethnicity, please specify	__________________________
A4	Tahap pendidikan tertinggi/ Highest educational level	[1] Tiada pendidikan formal/No formal education<br>[2] Sekolah rendah/ Primary school<br>[3] Sekolah menengah/ Secondary school<br>[4] Diploma/ Siji/ Diploma/Certificate/Skill (Post-secondary)<br>[5] Ijazah Muda/ Bachelor's degree<br>[6] Sarjana/ Ijazah Professional/ Master's/Professional degree<br>[7] Ijazah kedoktoran/ Doctorate degree
A5	Purata pendapatan bulanan/Monthly average income (RM/MYR)	[1] < RM1000<br>[2] RM1,001- RM3,000<br>[3] RM3,001-RM6,000<br>[4] RM6,001-RM9,000<br>[5] > RM9,000
A6	Pekerjaan/ Occupation	____________________________<br><br>[1] Professional dan pengurusan/ Professional and managerial<br>[2] Pekerja mahir/ Skilled worker<br>[3] Pekerja tidak mahir/ Non-skilled worker<br>[4] Pelajar/ Student

		[5] Surirumah/ Housewife<br>[6] Bersara/ Retired
A7	Yang manakah antara berikut menerangkan tempat anda tinggal/ Which of the following best describes the area you live in?	[1] Bandar/ Urban<br>[2] Pinggir bandar/ Suburban<br>[3] Kampung/ Rural
A8	Negeri/ State	[1] Johor<br>[2] Kedah<br>[3] Kelantan<br>[4] Melaka<br>[5] Negeri Sembilan<br>[6] Pahang<br>[7] Perak<br>[8] Perlis<br>[9] Pulau Pinang<br>[10] Sabah<br>[11] Sarawak<br>[12] Selangor<br>[13] Terengganu<br>[14] WP Kuala Lumpur<br>[15] WP Labuan<br>[16] WP Putrajaya

Bahagian B/ Section B<br><br>PENGETAHUAN TENTANG ANTIBIOTIK DAN KETAHANAN ANTIBIOTIK<br>KNOWLEDGE ABOUT ANTIBIOTIC AND ANTIBIOTIC RESISTANCE		
B1	Maklumat am tentang antibiotik/ General information about antibiotik	
B1.1	Demam dan selsema disebabkan oleh virus, bukannya bakteria/ The common cold and flu are caused by viruses, not by bacteria	[1] Betul/ True<br>[2] Salah/ False<br>[3] Tidak tahu/ Don’t know
B1.2	Antibiotik hanya efektif terhadap jangkitan bakteria/ Antibiotics are only effective against bacterial infections	[1] Betul/ True<br>[2] Salah/ False<br>[3] Tidak tahu/ Don’t know
B1.3	Antibiotik efektif terhadap jangkitan yang disebabkan oleh virus(demam atau selesema)/ Antibiotics are effective against infection caused by viruses (e.g. common cold or the flu)	[1] Betul/ True<br>[2] Salah/ False<br>[3] Tidak tahu/ Don’t know

B1.4	Antibiotik mempercepatkan proses penyembuhan dari demam dan selesema/Antibiotics speed up the recovery from most coughs and colds	[1] Betul/ True<br>[2] Salah/ False<br>[3] Tidak tahu/ Don’t know
B1.5	Antibiotik yang berbeza diperlukan untuk menyembuhkan penyakit yang berbeza/ Different antibiotics are needed to cure different diseases	[1] Betul/ True<br>[2] Salah/ False<br>[3] Tidak tahu/ Don’t know
B2	Penggunaan antibiotic/ Antibiotic use	
B2.1	Adalah tidak menjadi masalah untuk menggunakan lebihan antibiotik daripada rawatan lepas/ It is okay to use leftover antibiotics from previous treatments	[1] Betul/ True<br>[2] Salah/ False<br>[3] Tidak tahu/ Don’t know
B2.2	Adakah tidak menjadi masalah untuk menggunakanantibiotik yang diberikan oleh kawan-kawan atau ahli keluarga, selagi mana ianya digunakan untuk menyembuhkan penyakit yang sama/<br>It’s okay to use antibiotics that were given to a friend or family member, as long as they were used to treat the same illness	[1] Betul/ True<br>[2] Salah/ False<br>[3] Tidak tahu/ Don’t know
B2.3	Adalah tidak menjadi masalah untuk menyimpan antibiotic bagi kegunaan akan datang/ It’s okay to save antibiotics for later use	[1] Betul/ True<br>[2] Salah/ False<br>[3] Tidak tahu/ Don’t know
B2.4	Adalah tidak menjadi masalah untuk membeli antibiotic tanpa preskripsi daripada doctor/ It is okay to buy antibiotics without a prescription from a doctor	[1] Betul/ True<br>[2] Salah/ False<br>[3] Tidak tahu/ Don’t know
B2.5	Adalah tidak menjadi masalah untuk beli atau minta antibiotik yang sama daripada doktor, jika anda jatuh sakit dengan tanda-tanda yang sama, kerana antibiotik itu telah membantu anda sembuh sebelum ini/<br>It’s okay to buy or request the same antibiotics from a doctor, if you fall sick again with the similar symptoms as they helped you get better before”	[1] Betul/ True<br>[2] Salah/ False<br>[3] Tidak tahu/ Don’t know

B2.6	Saya sepatutnya berhenti mengambil antibiotik yang digunakan untuk rawatan saya apabila saya rasa bertambah sihat/ I should stop taking antibiotics used for my treatment when I feel better	[1] Betul/ True<br>[2] Salah/ False<br>[3] Tidak tahu/ Don’t know
B2.7	Saya sepatutnya berhenti mengambil antibiotik yang digunakan untuk rawatan saya apabila saya telah mengambil keseluruhan antibiotik seperti yang diarahkan oleh doktor/ I should stop taking antibiotics used for my treatment when I have taken the full course of antibiotics as directed by the doctor	[1] Betul/ True<br>[2] Salah/ False<br>[3] Tidak tahu/ Don’t know
B3	Penyakit yang boleh disembuhkan dengan antibiotic/ Diseases than can be treated with the antibiotics	
B3.1	Gonorea/ Gonorrhoea	[1] Betul/ True<br>[2] Salah/ False<br>[3] Tidak tahu/ Don’t know
B3.2	Jangkitan ginjal atausaluran kencing/Bladder infection orurinary tract infection (UTI)	[1] Betul/ True<br>[2] Salah/ False<br>[3] Tidak tahu/ Don’t know
B3.3	Cirit birit/ Diarrhoea	[1] Betul/ True<br>[2] Salah/ False<br>[3] Tidak tahu/ Don’t know
B3.4	Demam dan selesema/ Coldand flu	[1] Betul/ True<br>[2] Salah/ False<br>[3] Tidak tahu/ Don’t know
B3.5	Deman/ Fever	[1] Betul/ True<br>[2] Salah/ False<br>[3] Tidak tahu/ Don’t know
B3.6	Malaria/ Malaria	[1] Betul/ True<br>[2] Salah/ False<br>[3] Tidak tahu/ Don’t know
B3.7	Campak/ Measles	[1] Betul/ True<br>[2] Salah/ False<br>[3] Tidak tahu/ Don’t know

B3.8	Jangkitan kulit atau luka/ Skin or wound infection	[1] Betul/ True<br>[2] Salah/ False<br>[3] Tidak tahu/ Don't know
B3.9	Sakit tekak/ Sore throat	[1] Betul/ True<br>[2] Salah/ False<br>[3] Tidak tahu/ Don't know
B3.10	Sakit badan/ Body aches	[1] Betul/ True<br>[2] Salah/ False<br>[3] Tidak tahu/ Don't know
B3.11	Sakit kepala/ Headaches	[1] Betul/ True<br>[2] Salah/ False<br>[3] Tidak tahu/ Don't know
B4	Kesan sampingan antibiotic/ Antibiotic side effects	
B4.1	Antibiotik boleh menyebabkan alahan / Antibiotic can cause allergic reaction	[1] Betul/ True<br>[2] Salah/ False<br>[3] Tidak tahu/ Don't know
B4.2	Muntah dan mual/Vomiting and nausea	[1] Betul/ True<br>[2] Salah/ False<br>[3] Tidak tahu/ Don't know
B4.3	Cirit-birit/Diarrhoea	[1] Betul/ True<br>[2] Salah/ False<br>[3] Tidak tahu/ Don't know
B4.4	Sakit pada abdomen/ Abdominal pain	[1] Betul/ True<br>[2] Salah/ False<br>[3] Tidak tahu/ Don't know
B4.5	Reaksi pada kulit (gatal/ luka) / Skin reaction (rashes/ulcers)	[1] Betul/ True<br>[2] Salah/ False<br>[3] Tidak tahu/ Don't know
B4.6	Toksik pada hati/ Livertoxicity	[1] Betul/ True<br>[2] Salah/ False<br>[3] Tidak tahu/ Don't know
B4.7	Toksik pada buah pinggang/ Kidney toxicity	[1] Betul/ True<br>[2] Salah/ False<br>[3] Tidak tahu/ Don't know
B4.8	Jika anda mendapat kesan sampingan semasa mengambil antibiotik, anda sepatutnya berjumpa doctor secepat mungkin/	[1] Betul/ True<br>[2] Salah/ False<br>[3] Tidak tahu/ Don't know

	If you get side effects during a course of antibiotics treatment you should see your doctor immediately	
B4.9	Jika anda ada sejarah kesan sampingan atau alahan kepada antibiotic, anda perlu memaklumkan kepada doctor/ahli farmasi/<br>If you have a history of side effects or allergy to antibiotics, you should inform your doctor/pharmacist	[1] Betul/ True<br>[2] Salah/ False<br>[3] Tidak tahu/ Don't know
B4.10	Antibiotik boleh menyebabkan ketidakseimbangan kepada bakteria floral dalam badan /<br>Antibiotics can cause imbalance in the body's own bacterial flora	[1] Betul/ True<br>[2] Salah/ False<br>[3] Tidak tahu/ Don't know
B5	Ketahanan antibiotic/ Antibiotik	
B5.1	Pernahkah anda DENGAR mana-mana perkataan di bawah/ Have you HEARD of any of the following terms:	
B5.1.1	Ketahanan antibiotik/ Antibiotic resistance	[1] Betul/ True<br>[2] Salah/ False<br>[3] Tidak tahu/ Don't know
B5.1.2	Superbug/ Superbugs	[1] Betul/ True<br>[2] Salah/ False<br>[3] Tidak tahu/ Don't know
B5.1.3	Ketahanan antimicrobial/ Antimicrobial resistance (AMR)	[1] Betul/ True<br>[2] Salah/ False<br>[3] Tidak tahu/ Don't know
B5.1.4	Ketahanan ubat/ Drug resistance	[1] Betul/ True<br>[2] Salah/ False<br>[3] Tidak tahu/ Don't know
B5.1.5	Bakteria tahan antibiotik/ Antibiotic-resistant bacteria	[1] Betul/ True<br>[2] Salah/ False<br>[3] Tidak tahu/ Don't know
B5.2	Masalah ketahanan antibiotik/ Antibiotic resistance problem	
B5.2.1	Ketahanan antibiotik berlaku apabila badan menjadi tahan kepada antibiotik, di mana antibiotik tidak	[1] Betul/ True<br>[2] Salah/ False<br>[3] Tidak tahu/ Don't know

	lagi berkesan / Antibiotic resistance occurs when body becomes resistant to antibiotics, where antibiotic no longer and they no longer work as well	
B5.2.2	Banyak jangkitan yang menjadi tahan (tidak lagi berkesan) terhadap rawatan antibiotik/ Many infections are becoming increasingly resistant to treatment by antibiotics	[1] Betul/ True<br>[2] Salah/ False<br>[3] Tidak tahu/ Don't know
B5.2.3	Ketahanan antibiotik adalah isu yang boleh memberi kesan kepada saya dan generasi akan datang / Antibiotic resistance is an issue that could affect me and my future generation	[1] Betul/ True<br>[2] Salah/ False<br>[3] Tidak tahu/ Don't know
B5.2.4	Ketahanan antibiotik adalah satu isu di negara lain, bukannya disini (Malaysia) / Antibiotic resistance is an issue in other countries but not here	[1] Betul/ True<br>[2] Salah/ False<br>[3] Tidak tahu/ Don't know
B5.2.5	Ketahanan antibiotic hanyalah masalah kepada orang yang selalu mengambil antibiotik / Antibiotic resistance is only aproblem for people who take antibiotics regularly	[1] Betul/ True<br>[2] Salah/ False<br>[3] Tidak tahu/ Don't know
B5.2.6	Ketahanan antibiotik hanyalah masalah kepada orang yang selalu mengambil antibiotik / Antibiotic resistance is only a problem for people who take antibiotics regularly	[1] Betul/ True<br>[2] Salah/ False<br>[3] Tidak tahu/ Don't know
B5.2.7	Bakteria yang menjadi tahan kepada antibiotik boleh disebarkan daripada seseorang ke seseorang / Bacteria which are resistant to antibiotics can be spread from person to person	[1] Betul/ True<br>[2] Salah/ False<br>[3] Tidak tahu/ Don't know
B5.2.8	Anda boleh menjadi pembawa kepada bakteria tahan dan menyebarkannya kepada kawan dan/atau ahli keluarga / You can be a carrier of resistant bacteria and pass them to your friends and/or family members.	[1] Betul/ True<br>[2] Salah/ False<br>[3] Tidak tahu/ Don't know
B5.2.9	Jangkitan disebabkan ketahanan antibiotik boleh menyebabkan prosedur perubatan seperti	[1] Betul/ True<br>[2] Salah/ False<br>[3] Tidak tahu/ Don't know

	pembedahan, pemindahan organ dan rawatan cancer menjadi lebih bahaya/ Antibiotic-resistant infections could make medical procedures like surgery, organ transplants and cancer treatment much more dangerous	
B5.2.10	Jika anda sakit dan bakteria menjadi tahan kepada antibiotik yang telah dipreskripsi, penyakit anda boleh berterusan / If you become sick and your bacteria are resistant to your prescribed antibiotic, your illness could last longer	[1] Betul/ True<br>[2] Salah/ False<br>[3] Tidak tahu/ Don't know
B5.2.11	Mengambil antibiotic sewenang-wenangnya boleh menyumbang kepada ketahanan antibiotic /Taking antibiotics unnecessarily may contribute to the development of antibiotic resistance.	[1] Betul/ True<br>[2] Salah/ False<br>[3] Tidak tahu/ Don't know
B5.2.12	Melengkapkan pengambilan antibiotik adalah penting untuk sepenuhnya membunuh bakteria yang menyebabkan sakit / Complete the course of antibiotic prescribed is important to completely kill the bacteria that cause your illness	[1] Betul/ True<br>[2] Salah/ False<br>[3] Tidak tahu/ Don't know
B5.2.13	Penggunaan antibiotik dalam haiwan boleh mengurangkan kesan antibiotik di kalangan manusia / The use of antibiotics among animals can reduce the effect of antibiotics among humans	[1] Betul/ True<br>[2] Salah/ False<br>[3] Tidak tahu/ Don't know
B5.2.14	Haiwan boleh menjadi pembawa kepada bakteria tahan antibiotik dan menyebarkan kepada manusia / Animals can act as carriers of the resistant bacteria and pass them to human	[1] Betul/ True<br>[2] Salah/ False<br>[3] Tidak tahu/ Don't know

Bahagian C/ Section C<br><br>AMALAN PENGGUNAAN ANTIBIOTIK<br>PRACTICES OF ANTIBIOTIC USE		
C1	Amalan mendapatkan antibiotik/ Practices of obtaining antibiotic	
C1.1	Mendapatkan antibiotic di farmasi tanpa preskripsi /<br>Get antibiotics at the pharmacy without a prescription	[1] Tidak pernah/ Never<br>[2] Jarang-jarang/ Rarely<br>[3] Kadang-kadang/ Sometimes<br>[4] Kerap/ Often
C1.2	Minta pendapat doktor sebelum mula ambil antibiotik /<br>Consult a doctor before starting antibiotics	[1] Tidak pernah/ Never<br>[2] Jarang-jarang/ Rarely<br>[3] Kadang-kadang/ Sometimes<br>[4] Kerap/ Often
C1.3	Pergi kepada doctor lain sekiranya seorang doktor tidak mahu memberikan antibiotik /<br>Going to another doctor if a doctor refuses to give antibiotic	[1] Tidak pernah/ Never<br>[2] Jarang-jarang/ Rarely<br>[3] Kadang-kadang/ Sometimes<br>[4] Kerap/ Often
C2	Amalan menggunakan antibiotic dan pengambilan dos/ Practices of antibiotic consumption and dosing	
C2.1	Mengambil antibiotik seperti yang telah dipreskripsi doktor/ Consume the antibiotics as prescribed by your doctor	[1] Tidak pernah/ Never<br>[2] Jarang-jarang/ Rarely<br>[3] Kadang-kadang/ Sometimes<br>[4] Kerap/ Often
C2.2	Ambil antibiotik untuk demam biasa (hidung berhingus, sakit tekak, batuk) / Take antibiotic for the common cold (runny or stuffy nose, sore throat, cough)	[1] Tidak pernah/ Never<br>[2] Jarang-jarang/ Rarely<br>[3] Kadang-kadang/ Sometimes<br>[4] Kerap/ Often
C2.3	Sengaja menggunakan dos lebih rendah daripada yang disyorkan oleh doctor atau farmasi /<br>Intentionally using a lower dose of antibiotics than recommended by doctor or pharmacist	[1] Tidak pernah/ Never<br>[2] Jarang-jarang/ Rarely<br>[3] Kadang-kadang/ Sometimes<br>[4] Kerap/ Often
C2.4	Sengaja menggunakan dos lebih tinggi daripada yang disyorkan oleh doKtor atau farmasi / Intentionally use a higher dose of antibiotic than recommended by doctor or pharmacist	[1] Tidak pernah/ Never<br>[2] Jarang-jarang/ Rarely<br>[3] Kadang-kadang/ Sometimes<br>[4] Kerap/ Often
C2.5	Gagal untuk ikut dosyang	[1] Tidak pernah/ Never

	disyorkan(tertinggal dos, tidaksengaja terlebih dos) /<br>Fail to comply with the dosing recommendation (eg. missed dose, accidentally overdose)	[2] Jarang-jarang/ Rarely<br>[3] Kadang-kadang/ Sometimes<br>[4] Kerap/ Often
C2.6	Berhenti menggunakan antibiotik apabila tanda-tanda penyakit semakin baik/ Discontinue use of antibiotic when symptoms improve	[1] Tidak pernah/ Never<br>[2] Jarang-jarang/ Rarely<br>[3] Kadang-kadang/ Sometimes<br>[4] Kerap/ Often
C3	Amalan penggunaan lebihan antibiotik atau antibiotik yang tidak habis digunakan/ Practices of leftover/unfinished antibiotic	
C3.1	Beri lebihan antibiotik kepada haiwan peliharaan/ Give leftover antibiotics to your pets	[1] Tidak pernah/ Never<br>[2] Jarang-jarang/ Rarely<br>[3] Kadang-kadang/ Sometimes<br>[4] Kerap/ Often
C3.2	Gunakannya sebagai baja untuk pokok/ Use it as fertilizer for plants	[1] Tidak pernah/ Never<br>[2] Jarang-jarang/ Rarely<br>[3] Kadang-kadang/ Sometimes<br>[4] Kerap/ Often
C3.3	Kongsi lebihan antibiotik dengan orang lain/ Share leftover antibiotics with other people	[1] Tidak pernah/ Never<br>[2] Jarang-jarang/ Rarely<br>[3] Kadang-kadang/ Sometimes<br>[4] Kerap/ Often
C3.4	Buang dalam longkang/sistem perparitan<br>Throw it into a sewage/drainage system	[1] Tidak pernah/ Never<br>[2] Jarang-jarang/ Rarely<br>[3] Kadang-kadang/ Sometimes<br>[4] Kerap/ Often
C3.5	Guna lebihan antibiotik daripada rawatan yang lepas apabila tanda-tanda yang muncul /<br>Use leftover antibiotics from previous treatments when similar symptoms present	[1] Tidak pernah/ Never<br>[2] Jarang-jarang/ Rarely<br>[3] Kadang-kadang/ Sometimes<br>[4] Kerap/ Often
C3.6	Kembalikan kepada farmasi/klinik/mana-mana tetapan penjagaan kesihatan/<br>Return to the pharmacy/clinic/any healthcare setting	[1] Tidak pernah/ Never<br>[2] Jarang-jarang/ Rarely<br>[3] Kadang-kadang/ Sometimes<br>[4] Kerap/ Often
C3.7	Buang dalam sampah biasa /<br>Throw it in general waste	[1] Tidak pernah/ Never<br>[2] Jarang-jarang/ Rarely

		[3] Kadang-kadang/ Sometimes<br>[4] Kerap/ Often

---TAMAT/END---
-
