# Supplementary figures and images for: Factors influencing inappropriate use of antibiotics: Findings from a nationwide survey of the general public in Malaysia
Source: PLoS One. 2021 Oct 20;16(10):e0258698. doi: 10.1371/journal.pone.0258698 (PMC8528291; doi:10.1371/journal.pone.0258698)

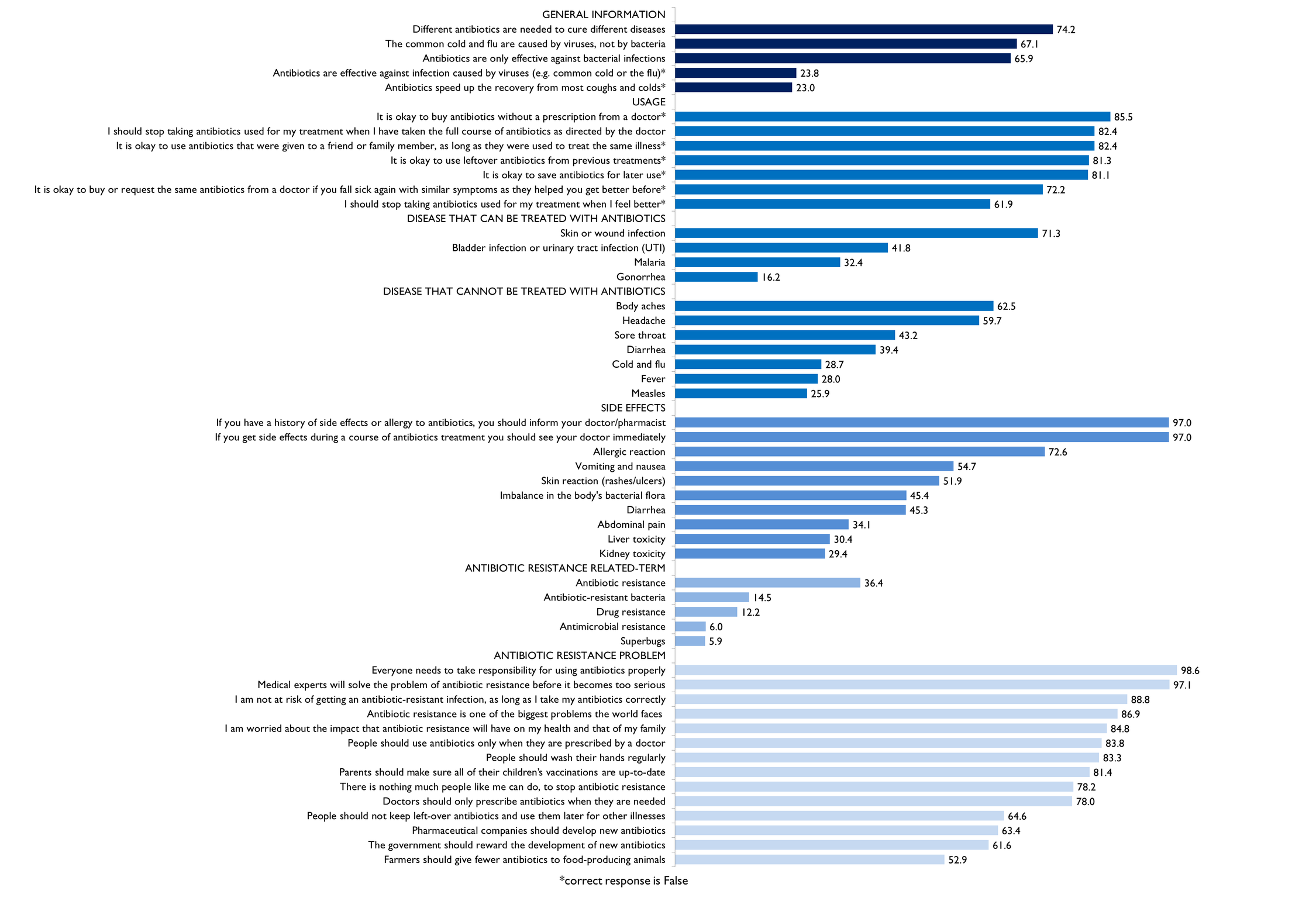

Supplement: S1 Fig — (TIF) [file pone.0258698.s002.tif]

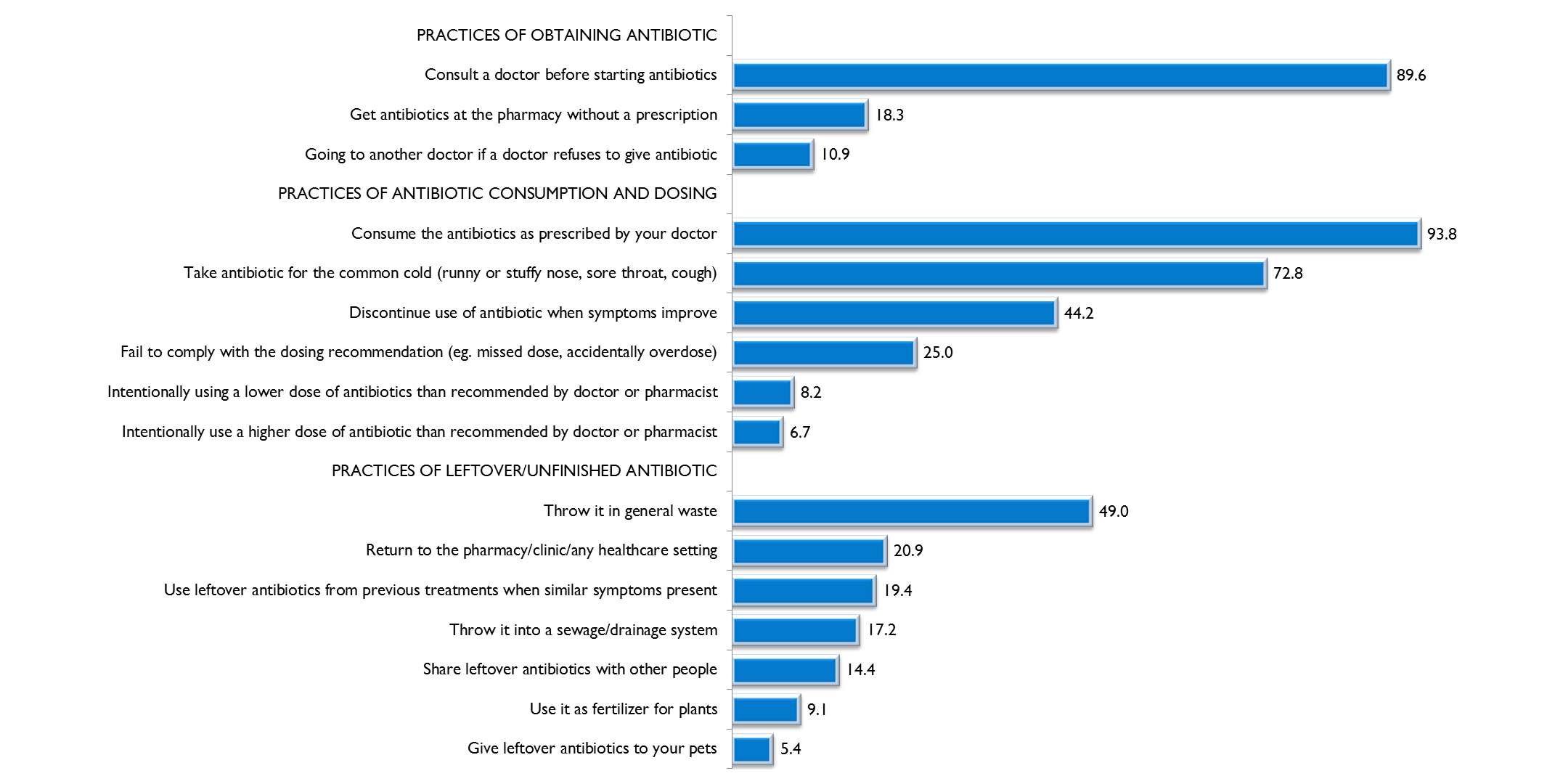

Supplement: S2 Fig — (TIF) [file pone.0258698.s003.tif]
