# Supplementary material for: Factors influencing inappropriate use of antibiotics: Findings from a nationwide survey of the general public in Malaysia
Source: PLoS One. 2021 Oct 20;16(10):e0258698. doi: 10.1371/journal.pone.0258698 (PMC8528291; doi:10.1371/journal.pone.0258698)
Supplement: Attachment — Submitted filename: #Response to reviewers 29July2021.docx [file pone.0258698.s004.docx]

**Comments to the Author**

1. Is the manuscript technically sound, and do the data support the conclusions?

The manuscript must describe a technically sound piece of scientific research with data that supports the conclusions. Experiments must have been conducted rigorously, with appropriate controls, replication, and sample sizes. The conclusions must be drawn appropriately based on the data presented.

Reviewer #1: Partly

Reviewer #2: Partly

2. Has the statistical analysis been performed appropriately and rigorously?

Reviewer #1: No

Reviewer #2: No

3. Have the authors made all data underlying the findings in their manuscript fully available?

The [PLOS Data policy](http://www.plosone.org/static/policies.action#sharing) requires authors to make all data underlying the findings described in their manuscript fully available without restriction, with rare exception (please refer to the Data Availability Statement in the manuscript PDF file). The data should be provided as part of the manuscript or its supporting information, or deposited to a public repository. For example, in addition to summary statistics, the data points behind means, medians and variance measures should be available. If there are restrictions on publicly sharing data—e.g. participant privacy or use of data from a third party—those must be specified.

Reviewer #1: No

Reviewer #2: No

4. Is the manuscript presented in an intelligible fashion and written in standard English?

PLOS ONE does not copyedit accepted manuscripts, so the language in submitted articles must be clear, correct, and unambiguous. Any typographical or grammatical errors should be corrected at revision, so please note any specific errors here.

Reviewer #1: Yes

Reviewer #2: No

5. Review Comments to the Author

Please use the space provided to explain your answers to the questions above. You may also include additional comments for the author, including concerns about dual publication, research ethics, or publication ethics. (Please upload your review as an attachment if it exceeds 20,000 characters)

Reviewer #1: 1. The actual questionnaire is not available.
Reply: Questionnaire uploaded in submission.

2. This is a newly developed questionnaire but the survey questions were only partially validated. I suggest the authors assess the validity and reliability of the questionnaire by test-retest assessment, inter-rater analysis, using principal component analysis, then check the internal consistency of questions loading onto the same factor.
Reply: Thank you for suggestions. To carry out additional validity and reliability of the questionnaire by test-retest assessment, inter-rater analysis, using principal component analysis will be a great deal of work involving administer questions on additional study subject (for test-retest and inter-rater analyses), and is beyond the scope of this study. Extensive validity and reliability reporting usually can be a full manuscript, which is fully focus on reporting the psychometric properties of a scale. The aim of our study is not to develop a tools for measurement of knowledge of antibiotic use and antibiotic resistance, therefore we only carry out internal consistency check.

Nonetheless, we report this in the limitation section.

Line 354 (Note: Line number referring to the tracked version))

The third limitation is the use of self-developed scale for measurement of antibiotic and antibiotic resistance-related knowledge. Only the internal consistency of the scale was examined. Extensive psychometric analyses were not carried out. Therefore, the measurements using these scales should be interpreted with cautiously.

3. Please state the supporting resources provided by the WHO.
Reply: We added the detail supporting resources by WHO, nonetheless we removed from the manuscript as requested, and indicated the source in the submission system.

Line 370-371: This study was supported by World Health Organization (WHO). Purchase Order 202241929. WHO Registration 2018/903873-0.

4. "Sg Buloh Hopsital" was misspelled. It should be "Sg. Buloh Hospital".

Amended: Line 8 Sg.

5. The introductory sentence in the Abstract is misleading, readers may think that this study is assessing the threat of antimicrobial resistance in Malaysia.
We rephrased the Introduction section in abstract

Line 21-22: Antibiotic resistance is one of the biggest threats to global public health. Misuse of antibiotics has never been investigated on a nationwide scale in Malaysia.

6. In the "Introduction" section, reference 5 (2013) is out of date. Please provide updated information.

Replace, Line 53-57. According to the Centres for Disease Control and Prevention (CDC), an estimate of 2.8million people are infected with antibiotic-resistance bacteria or fungi, and more than 35,000 people die as a result [5].

Centers for Disease Control and Prevention. [Antibiotic / Antimicrobial Resistance (AR / AMR)](https://www.cdc.gov/drugresistance/index.html). 2020. [ Cited 2021 July 24]. Available from https://www.cdc.gov/drugresistance/about.html

7. In the "Instruments" section, individuals may interpret “rarely”, “occasionally”, “sometimes”, “often” differently. The scales between “rarely”, “occasionally”, “sometimes”, “often” are not linear. How did the researchers address this inter-rater or “inter-respondent” difference?
Reply: As noted, we reported internal consistency of all the items in our scale, internal consistency will include consistency of responses of the option answer “rarely”, “occasionally”, “sometimes”, “often”.

Nonetheless, we did not specifically carry out investigation on “inter-respondent” differences. We noted this in Limitation section.

Line 355: Only the internal consistency of the scale was examined. Extensive psychometric analyses were not carried out. Therefore, the measurements using these scales should be interpreted cautiously.

8. There are 5 response options in the practices of antibiotic use questions; namely, never, rarely, occasionally, sometimes, and often. How are the 4 score options of 0, 1, 2, and 3 awarded to 5 response options?

Reply: Corrected, there are four option answer

Rephrased, Line 117. For each question, the response options were *never*, *rarely*, *sometimes*, and *often*, scored as 0, 1, 2, and 3, respectively.

9. The "Statistical analyses" section is not clear. The authors may describe clearly how the respondents were divided into 2 groups for comparisons.

Added line 154-156: The outcome variable level of knowledge and antibiotic practices in the multivariate analyses we divided into two groups for comparison based on the median split.

10. In 2019, the average monthly household income in Malaysia is RM 7,901. About 75% of the respondents in the study fall under the bottom 20% of the classification of household income of the Malaysian community. Would the respondents be representative of a general Malaysian population?

We added this in the limitation section.

Line 358-361: Lastly, despite this study is a nationwide survey, the income level of the majority of our study respondents was below the average monthly household income of the Malaysian population. Of note, Malaysia’s median income was recorded at RM5,873 in the year 2019 [32]. Hence, our study may have a slight representation of respondents from the lower-income groups.

11. The racial distributions in Malaysia are Malays and indigenous peoples 62%, Chinese 20.6%, and Indian 6.2%. How well is the racial distribution of Malaysia reflected in the racial distribution of the respondents in this survey?

We added this in the limitation section.

Line 362: Additionally, the ethnic group distribution in our study is slightly different from the general Malaysian population, of which 67.4% were Malays and Bumiputera, 24.6% Chinese, 7.3% Indians, and 0.7% Others [33].

12. The official retirement age in Malaysia is 60 years. Is there any particular reason that the age of the respondents was categorized as 18-30, 31-50, and 51-89?

Reply: We received relatively lower proportion of respondents of age above 51 years, therefore, participants of age above 50 years old were grouped as one category.

13. What is the rationale for categorizing "students", "retired", "unemployed" as a group while they have a diverse age range, income, and experience?

Reply: “students", "retired", "unemployed"- these group is considered as group not in labor force, which is commonly categorized as a group, usually the number in this group is relatively small and is often categorized as one group in many studies.

14. Table 1, last section, the label for data "154 (37.4) p<0.001 Reference" is missing.. Table 1, last section, the label for data "154 (37.4) p<0.001 Reference" seems missing.

Reply: Reference was noted.

15. In the "Discussion" section, the authors state that better knowledge was found to be associated with more appropriate use of antibiotics. There seems to be a mismatch between the knowledge scores and the practice scores especially among the Indian race. The Indians who had a reasonable score (47% attaining a knowledge score range of 23-50) had very low practice scores (92% with a scores range of 17-39). Does it imply that an arbitrary cut-off point at the median score does not reflect the true underlying picture? Or Are the knowledge questions truly measure what they are supposed to measure?

Reply: The statement “The finding of better knowledge associated with more appropriate use of antibiotics” was of overall participants based of the multivariate model, as we reported in the manuscript. There are other factors (demographics and their interaction) taken consideration in the modelling. Therefore, it is not correct to compare the univariate results [ “The Indians who had a reasonable score (47% attaining a knowledge score range of 23-50) had very low practice scores (92% with a scores range of 17-39] with the multivariate findings of all participants, and furthermore the model include many other factors. It is also clear that the Indian represent a small proportion of study participants, therefore comparison with overall findings is not appropriate. This we viewed is not due to the cut-off point used that does not reflect the true underlying picture.

16. Also, different individuals have different perceptions on the terms rarely, occasionally, sometimes, and often. The “distance” or “scales” between these items are very arbitrary and not constant. Would the authors discuss quantifying these terms with a continuous number?
Reply: The scale is standard 4-point likert scale in ascending order. Quantifying the item into scores is often a practice in many social behavioral surveys, and certainly is may be subjected to bias in reporting. The bias in self-report and self-developed scales are justified in the Limitation section.

17. The resolution of Figure 1 and Figure 2 is poor.
We upload higher resolution Figure 1 and Figure 2.

18. There are some minor typographical and grammatical errors in the manuscript.

Reply: We amended the typographical and grammatical errors.

Line 54: estimated

Line 208: educational

Line 209: deleted “from”

Line 211: added “a”

Line 237: added “a”

Line 250: antibiotics

Line 253: were

Line 266: been heard

Line 271: the rational

Line 272: time to implement a campaign

Line 299: to

Reviewer #2: Thank you for giving me the chance to review this ms. Here are my comments:
1. authors have noted that - This study has documented important gaps in knowledge on antibiotic malpractices among
the Malaysian public - in my opinion it is not new! even the refs 9-13 have indicated that others have done such studies.

Reply: We noted that the our study is a larger scale that include nationwide sample.

Line 72-75: There have been several small*-*scale studies conducted in Malaysia, all of which suggest that local communities exhibit gaps in antibiotic awareness and appropriate antibiotic use [9-11]. Nonetheless, large-scale population-level data are lacking.

2. The Centre for Epidemiology and Evidence-Based Practice, Department of Social and Preventive Medicine should put its energy, money, time and effort to study the approach to improve and coming out with evidence-based interventions e.g. cost-effective interventions. The consequences and reasons for misuse are well documented, including in LMICs. So, what is the benefit of studying again and again ? We should find ways to overcoming these problems.

Reply: Unfortunately, the study objective of this manuscript is to determine KAP at a national level. Finding a way to overcome antibiotic misused is not the scope of this manuscript.

We noted this issues as future directions

Line 345-347

Future research is warranted to determine the effectiveness of antibiotic use intervention as well as to measure its cost-effectiveness to provide insights into effective strategies to promote prudent use of antimicrobials in the country.

3. Methods - are poorly written and structured. study design? sample size? sampling from the 16 areas? Malaysian is a multiracial society with different culture, belief and socio-economic status - are these factors been taken into consideration during sampling? analysis? any confounders?

Reply: We enhanced our methodology write-up.

Line 81: We conducted a cross-sectional nationwide survey of the general population in Malaysia.

Although Malaysia is a multiracial society with different culture, belief and socio-economic status, our sampling was convenience sampling method, the sample size was multiplied by the predicted design effect of two.

We added sample size calculation.

Line 85-90: The sample size was calculated using the equation: n = Z2 p(1-p)/d2. Using a margin of error of 0.05 (5%), with a 95% CI and 50% response distribution, the calculated sample size was 384. Due to the use of convenience sampling, the sample size was multiplied by the predicted design effect of two. Therefore, the minimum survey sample size was set to 768 (384 x 2) participants.

We carried further mediation analyses as suggest and added in Methodology and report the results

Line 160: We conducted causal mediation analysis to investigate the effect of confounder bias on the casual inference of the mediator to the outcome variables using regression analysis.

Line 240-242: The result of mediation analysis showed that age mediate the association between knowledge and antibiotic practices (β = 0.251; 95% CI: (0.131, 0.221).

Line 341-345: The result of mediating effect of age on the association between knowledge and antibiotic practices along with the finding of higher odds of positive antibiotic practices score in older age participants imply that individual of younger age should be the target population for antibiotic use interventions.

4. How effective is tel interview in Malaysia in getting a comprehensive views of the respondents?

Added line 90-91: Based on an estimate of 10% response rate of telephone survey [12], approximately 11,000 phone numbers were randomly selected from the telephone directory.

5. Inclusion criteria - e.g. had heard - if just heard but have not used before, do you think there will be a different in their responses, understanding, knowledge and practice?

Reply: The decision to use “had heard” was agreed by panel of expert in the research team and WHO team. Having heard of the term implies the person is able to comprehend our questions and respond to our survey.

We are not sure if the responses will be different in understanding, knowledge and practices, if we asked have “ever used”, this issue may need further study.

6. Tools and items - source of these items and questions? Your own ideas? or from the literature?

Reply: We developed the questions based on inputs of panels.

Line 122: All the questions were developed and validated by a panel of experts that consisted of physicians, academicians, and veterinarians from government agencies.

7. Translations - the process of translations? validations? lack of explanation, vague

We rephrased to enhance clarity.

Line 123: The questionnaire was developed in English. As the largest group of Malaysians consists of three main ethnicities, namely the Malays, Chinese, and Indians, the questionnaire was translated into Bahasa Malaysia (the national language of Malaysia), Chinese (Mandarin), and Tamil by native-speaking translators. The translated questionnaires were reviewed by independent translators and back translation was conducted on the primary translated version.

8. Interviewers - was the interrater reliability measured? how did you reduce potential bias?

Unfortunately, we did not carry out interrater reliability of different interviewer. However we conducted training on our interviewers to reduce potential bias.

Line: 132: The interviewers were trained to reduce interviewer related errors by ensuring that all respondents were asked identically worded questions without unscripted commentary that could bias response.

9. Cronbach's alpha - move to instrument development section

Reply: Moved to Line 119-121.

10. Analysis section - lack of information - univariate analysis? descriptive stats? how was the factors decided into the model, i.e. multivariate analysis?

Added Line 140: Descriptive statistics were used to describe the proportion of knowledge and antibiotic practices.

Line 156-158: In the multivariable logistic regression analyses, all variables found to have a statistically significant association (two-tailed, *p*-value < 0.05) in the univariate analyses were entered into the model via forced-entry method.

11. all % must be supplied with n i.e. (n, %)

Added Line 171-174.

The study had a slightly higher representation of female participants (67.8%, n=586), participants with tertiary education attainment (49.5%, n=428), and with an average monthly household income of MYR1001 to 3000 (47.8%, n=413). By ethnicity, the majority were Malay (51.5%, n=445).

Added 189- 197.

Figure 1 shows the correct responses of knowledge items. The majority had good knowledge of antibiotic usage and antibiotic resistance problems, where over 50% of participants provided correct responses on the question items. A small proportion was aware that antibiotics are not effective against infection caused by viruses (23.8%, n=206) and that antibiotics cannot speed up recovery from coughs and colds (23.0%, n=199). Additionally, a small proportion was aware that cold and flu cannot be treated by antibiotic (28.7%, n=248). Awareness of terms such as ‘drug resistance’ (12.2%, n=105), ‘antimicrobial resistance’ (6.0%, n=52), and ‘superbugs’ (5.9%, n=51) was extremely low. Knowledge regarding diseases that can or cannot be treated with antibiotics was also poor.

Added 215-223

Figure 2 shows the findings of practices relating to antibiotic use. Regarding practices of obtaining antibiotics, a small proportion reported ever obtaining antibiotics from a pharmacy without a physician prescription (18.3%, n=158) and seeking alternative doctors in obtaining antibiotics if a doctor refused to prescribe antibiotics (10.9%, n=94). Malpractice in adherence to recommended doses (25.0%, n=216) and using a lower (8.2%, n=71) or higher (6.7%, n=58) antibiotic dose than recommended were reported. A considerable proportion reused leftover antibiotics from previous treatments (19.4%, n=168), discarded leftover antibiotics (17.2%, n=149), shared leftover antibiotics with other people (14.4%, n=124), or gave leftover antibiotics to pets (5.4%, n=47).

12. Your age category - 51-89 - in this category - how do you explain and separate the elderly population's knowledge and practice?

Reply: We have very limited number of elderly participants, therefore we do not define our findings on the “elderly” , we reported the age group 51-89 as the “older age group participants”

13. Discussion - what does the study add? what are the study findings implication?

Line 337-338: This study adds to the evidence that better knowledge increased appropriate use of antibiotics.

There are several implication of the study as noted earlier in our first submission

Line 339-341: As antibiotic use is closely related to knowledge, our findings call for communication interventions that target the general public to fill the knowledge gaps identified in this study.

We also noted the future

Line 330-334: These disparities imply that there is a need to understand if the knowledge, attitudes, and practices of antibiotic use are deeply rooted in a sociocultural milieu that varies from one ethnic community to another. However, it should be noted that the number of Chinese and Indian participants in this study is relatively smaller than the Malays. There is a need to further explore this in future research.

Added in Conclusion.

Line 369-370: This implies that enhancing health literacy associated with antibiotic use, knowledge and awareness of antimicrobial resistance is a public health priority.

We noted implication in Conclusion in the first submission.

Line 374-376: These findings will help health policymakers in Malaysia to implement target-specific education interventions to enhance antimicrobial resistance health literacy and practices.

14. Figures 1 and 2 are blur and cannot be read

Reply: We provided a higher resolution Figure 1 and 2

15.This ms is better to be submitted in a local journal.

6. PLOS authors have the option to publish the peer review history of their article ([what does this mean?](https://journals.plos.org/plosone/s/editorial-and-peer-review-process#loc-peer-review-history)). If published, this will include your full peer review and any attached files.


If you choose “no”, your identity will remain anonymous but your review may still be made public.


**Do you want your identity to be public for this peer review?** For information about this choice, including consent withdrawal, please see our [Privacy Policy](https://www.plos.org/privacy-policy).

Reviewer #1: **Yes:** YokeLin LO

Reviewer #2: No

 ****************************************************************

**Response to email dated 29 July 2021**

We've checked your submission and before we can proceed, we need you to address the following issues:

1. Please amend your current ethics statement to address the following concerns: 
a) Did participants provide their written or verbal informed consent to participate in this study?

Rephrased line 129-130: Participation was voluntary and the participants provided verbal informed consent before the start of the telephone interview.

b) As consent was verbal, please explain i)  how you documented participant consent, and ii) whether the ethics committees/IRB approved this consent procedure.

Unfortunately during the data collection, we did not document/note the participant consent in paper. Once participant provided verbal consent, the enumerators proceed with interview. Those who did not consented will not proceed the interview session. Upon completion of interview implies consent, hence, this is the only means of documentation that we have.

Line 131: They were also informed that completion of the telephone interviewed implied consent to participate.

Added line 133-135: Informed consent was obtained verbally before the commencement of the telephone interview. This method of consent was approved by the ethics committee.

2. Thank you for stating the following financial disclosure:
“This study was supported by World Health Organization (WHO)”

Please state what role the funders took in the study.  If the funders had no role, please state: "The funders had no role in study design, data collection and analysis, decision to publish, or preparation of the manuscript."
If this statement is not correct you must amend it as needed.

Please respond by return e-mail so that we can amend your financial disclosure and competing interests on your behalf.

Reply. Our funder indeed had no role in study design, data collection and analysis, decision to publish, or preparation of the manuscript.

3. We note that the grant information you provided in the ‘Funding Information’ and ‘Financial Disclosure’ sections do not match.
When you resubmit, please ensure that you provide the correct grant numbers for the awards you received for your study in the ‘Funding Information’ section.

Noted, we have amend according.

4. Please upload a new copy of Figures 1 and 2 as the detail is not clear. Please follow the link for more information: <https://blogs.plos.org/plos/2019/06/looking-good-tips-for-creating-your-plos-figures-graphics/>" <https://blogs.plos.org/plos/2019/06/looking-good-tips-for-creating-your-plos-figures-graphics>.

Reply: We have redo the Figures and checked the criteria of our Figures in the following link <https://pacev2.apexcovantage.com/> , and have been approved. However, please let us know if the figures are still unclear, we may need to redo and split them into sub-figures with the figure.

5. Thank you for stating the following in the Acknowledgments/Funding  Section of your manuscript:
“This study was supported by World Health Organization (WHO). Order 202241929. WHO Registration 2018/903873-0”

We note that you have provided funding information that is not currently declared in your Funding Statement. However, funding information should not appear in the Acknowledgments section or other areas of your manuscript. We will only publish funding information present in the Funding Statement section of the online submission form.

Noted, we removed funding information from the manuscript

Please remove any funding-related text from the manuscript and let us know how you would like to update your Funding Statement. Currently, your Funding Statement reads as follows:
 “This study was supported by World Health Organization (WHO)”
