# Supplementary material for: Factors influencing inappropriate use of antibiotics: Findings from a nationwide survey of the general public in Malaysia
Source: PLoS One. 2021 Oct 20;16(10):e0258698. doi: 10.1371/journal.pone.0258698 (PMC8528291; doi:10.1371/journal.pone.0258698)
Supplement: Attachment — Submitted filename: Response to reviewers comments.docx [file pone.0258698.s005.docx]

Reviewers' comments:

Reviewer's Responses to Questions

**Comments to the Author**

1. If the authors have adequately addressed your comments raised in a previous round of review and you feel that this manuscript is now acceptable for publication, you may indicate that here to bypass the “Comments to the Author” section, enter your conflict of interest statement in the “Confidential to Editor” section, and submit your "Accept" recommendation.

Reviewer #2: All comments have been addressed

Reviewer #3: (No Response)

Reviewer #4: All comments have been addressed

2. Is the manuscript technically sound, and do the data support the conclusions?

The manuscript must describe a technically sound piece of scientific research with data that supports the conclusions. Experiments must have been conducted rigorously, with appropriate controls, replication, and sample sizes. The conclusions must be drawn appropriately based on the data presented.

Reviewer #2: Yes

Reviewer #3: Yes

Reviewer #4: Yes

3. Has the statistical analysis been performed appropriately and rigorously?

Reviewer #2: Yes

Reviewer #3: Yes

Reviewer #4: Yes

4. Have the authors made all data underlying the findings in their manuscript fully available?

The [PLOS Data policy](http://www.plosone.org/static/policies.action#sharing) requires authors to make all data underlying the findings described in their manuscript fully available without restriction, with rare exception (please refer to the Data Availability Statement in the manuscript PDF file). The data should be provided as part of the manuscript or its supporting information, or deposited to a public repository. For example, in addition to summary statistics, the data points behind means, medians and variance measures should be available. If there are restrictions on publicly sharing data—e.g. participant privacy or use of data from a third party—those must be specified.

Reviewer #2: Yes

Reviewer #3: Yes

Reviewer #4: Yes

5. Is the manuscript presented in an intelligible fashion and written in standard English?

PLOS ONE does not copyedit accepted manuscripts, so the language in submitted articles must be clear, correct, and unambiguous. Any typographical or grammatical errors should be corrected at revision, so please note any specific errors here.

Reviewer #2: Yes

Reviewer #3: Yes

Reviewer #4: Yes

6. Review Comments to the Author

Please use the space provided to explain your answers to the questions above. You may also include additional comments for the author, including concerns about dual publication, research ethics, or publication ethics. (Please upload your review as an attachment if it exceeds 20,000 characters)

Reviewer #2: Thank you for taking seriously the reviewers' comments and suggestions. The revised ms has improved its quality.

Reviewer #3: Title: Factors influencing inappropriate use of antibiotics findings from a nationwide survey in Malaysia

I would like to thank the Editor for giving me the opportunity to review this manuscript.
Overall, it is a well written manuscript and addresses an important area of medicine use. My specific comments are mentioned below:

Title: I suggest adding ‘general public’ in the title

Reply: Added “general public”

New title is “Factors influencing inappropriate use of antibiotics: findings from a nationwide survey of the general public in Malaysia”

Abstract:
Line 21, Misuse of antibiotic………, is not correct. There are other studies (<https://bmcinfectdis.biomedcentral.com/articles/10.1186/s12879-016-1530-2>). Hence I suggest authors to modify this sentence.

We do agree that there have been many studies on antibiotic misuse and practices, but all the studies were small scale studies. In contrast the current study is a nationwide sampling of all states in Malaysia.

We rephrase the sentence, line 22.

⇒Misuse of antibiotics has never been investigated on a nationwide scale among the general public in Malaysia.

Added in line 73

⇒ Furthermore, most of these studies have been limited by small sample size.

Line 32, ‘low awareness’ what do authors mean by this? These should be a cutoff score mentioned to categorize as ‘low’, ‘average’, ‘high etc.’.

Rephrase, line 33

⇒The proportions that were aware of the terms ‘drug resistance’, ‘antimicrobial resistance’, and ‘superbugs’ were low.

Introduction:
This section is generally well written. However, it lacks thorough literature review. I suggest authors to present more literature from Malaysia and from other countries.
A thorough justification for the research is lacking. Authors should describe similar studies from Malaysia and justify why this research was needed.
This section should add information for international readers. What it adds to international readers?
Line 61-63 shows similarity with <https://www.hindawi.com/journals/ipid/2018/8492740/>?
I suggest authors to run similarity check and modify it.

We have rephrased the sentence. Line 61

⇒ Various forms of inappropriate use of antibiotics, such as incomplete of entire course of antibiotic treatment, skipping doses, using leftover antibiotics from previous treatment, misuse of antibiotics to cure viral infections, and self-medication or purchase of antibiotics without prescription have been commonly reported [8].

Materials and methods:

The study timeline should be mentioned. Was it conducted during COVID 19 pandemic? Was there any stratification for samples? How do we ensure the samples are equally distributed in terms of gender, education, income, ethnicity, geographic distribution etc. I could see the females are much more than males? Can this influence the study findings? Do the selected samples reflect Malaysian population nationwide? If not this should be mentioned as a study limitation.

Reply

We mentioned in line 81 that Telephone interviews were conducted between June 2019 and Dec 2019, study is not conducted during COVID-19 pandemic.

There was no stratification for samples, the study is a cross sectional study, we noted in Line 81

“We conducted a cross-sectional nationwide survey of the general population in Malaysia. Telephone interviews were conducted between June 2019 and Dec 2019 using a computer-assisted telephone interview (CATI) system. The telephone numbers were randomly generated by computer from the latest electronic residential telephone directory (2018/2019) of all 13 states and 3 Federal Territories in Malaysia to obtain a representative sample.

The limitation of cross-sectional study was note din limitation, we further added the following, line 364.

⇒Lastly, despite this study is a nationwide survey that collected data from all states in Malaysia, some aspect of the cross-sectional design limit the generalization of our findings. The response rate was higher in females compared to males.

Instruments
Line 109, the knowledge scores should be categorized as low, average, and high. What is the cutoff score to category as ‘high’? The same has to be done for practice scores also. It would be more logical to divide the scores as low, average and high.

As the knowledge score is not normally distributed, the scores was divided into two range, based on median split, with higher score range indicates higher level of knowledge and appropriate practices. In the event the scores were divide into three range as low, average and high, the total with the lower score range was too small.

Added in line 206 for knowledge score

⇒Higher score range indicates a higher level of knowledge.

Added in line 234 for practices scores

⇒Higher score range indicates a higher level of appropriate antibiotic practices.

Line 117-119 is not enough. A more detailed description on questionnaire development should be added. Did authors obtain some information from published literature? In that case those should be cited.

Line 122
⇒All the questions were developed in reference to previous literature and guideline [13,14] by the research team and validated by panel of experts that consisted of physicians, academicians, and veterinarians from government agencies.

Line 92-96, shows similarity with <https://link.springer.com/article/10.1186/s12889-016-3409-y>
Authors copied verbatim and hence needs to be edited.

Rephrased: Line 92

⇒In each contacted household, only one person was surveyed. If more than one person in the contacted household met the inclusion criteria, one person was randomly selected to respond to the survey using. A random number table was used to select the participant to be included in the survey. To avoid over-representation of unemployed participants, all interviews were conducted between 5:30 pm and 10:00 pm on weekdays and from 12:00 p.m. to 7:00 p.m. on weekends or public holidays. Attempts to call at least two times on separate days we made for unanswered calls in the contacted household before being regarded as non-responses.

Results:

Line 181, How did authors arrive at ‘high scores’ ? How were the scores categorized?

Median split to form high and low score groups, hence added in line 151

Dichotomization of total knowledge and practices scores were done using median split to form high and low score groups [15].

Line 191, On what basis did authors perform mean and median scores? Did authors perform a normality check? If yes, mention the name of the test with p value in the methodology section.

Added in methodology, line 150

⇒The normality of total knowledge and practices scores were checked using the Kolmogorov-Smirnov test.

Added

⇒ On checking the normality distribution of the antibiotic knowledge score using the Kolmogorov-Smirnov test, it was found that the data were not normally distributed (*p <*0.05).

Line 194-195, this information should be mentioned in the methodology and references should be provided for median split.

Added line 151

⇒Dichotomization of total knowledge and practices scores were done using median split to form high and low score groups [15].

Line 217, was the normal distribution performed for practice scores?

Added Line 232

⇒Likewise, the significant value of the Kolmogorov-Smirnov test was less than 0.05 implies the data were not normally distributed.

Discussion:
This section is well written. It can be further improvised by discussing more on policy recommendations based on the research findings.

Line 300

⇒More stringent inspections by the regulatory authorities are needed to avoid dispensing antibiotics without a prescription.

Line 324

⇒ A policy aimed at preventing the disposal of antibiotics as household waste should be developed and implemented.

Line 328

⇒ Although some hospitals may have their own guidelines [30], there is a lack of national guidance on return unwanted medications. In 2010, the Pharmaceutical Services Division, Ministry of Health Malaysia (MOH) implemented the Return Your Medicines Program encouraging patients to return their unused or excess medicines to the pharmacy counter or medicine return box provided at the pharmacy facilities in the MOH hospitals and health clinics [31]. Patients should be informed and made aware of such guidelines so that they know where to return their expired, unwanted, or unused antibiotics.

Lines 241- 255. There is nothing new in this part. Antibiotics in viral infections is a well-known fact.

Reply : We noted that our study “in concordance with previous small-scale findings in Malaysia ..” Although nothing new, this implies the validity of our findings which is also logically similar to other smaller scale studies.

Line 254, …..public’s antibiotic stewardship behavior’ needs amendment. Try to rephrase with more suitable words.

Rephrased “appropriate use of antibiotics”

Limitations:
Line 340, Strengths and limitations can be in two different paragraphs.

Our strength is only in one sentence, hence it is placed as the same paragraph after limitation.

Despite these limitations, the study was the first nationwide survey in Malaysia to evaluate antibiotic knowledge and practices that was carried out on a wide spectrum of socio-demographics.

Line 356, I suggest authors to perform thorough literature review and see whether their claim is right.

Reply: We have performed thorough literature review and confirm the accuracy.

Figure 1, I had problem in viewing the Figure 1. The font size is too small. I suggest authors to check this.

Reply: We resubmitted Figure 1

Reviewer #4: Authors have article on Knowledge about antibiotic use and misuse using telephonic interviewing Malaysia . Due to widespread antibiotic misuse antibiotic resistance is increasing & multi drug resistant pathogen are common .
Topic is very relevant and worth studying and results show lack knowledge among low socioeconomic strata & educational level as reason for decreased awareness.
Study was done during COVID Pandemic whether Pandemic has affected survey results? Any specific points of lockdown related rise in misuse=se & stress related response is not clear. This can be clarified.

The study is not conducted during COVID-19 period

Telephonic survey results could be inferior to personal questionnaire based study

Justify the limitation of telephone survey in Limitation.

7. PLOS authors have the option to publish the peer review history of their article ([what does this mean?](https://journals.plos.org/plosone/s/editorial-and-peer-review-process#loc-peer-review-history)). If published, this will include your full peer review and any attached files.


If you choose “no”, your identity will remain anonymous but your review may still be made public.


**Do you want your identity to be public for this peer review?** For information about this choice, including consent withdrawal, please see our [Privacy Policy](https://www.plos.org/privacy-policy).

Reviewer #2: **Yes:** Mohamed Izham Mohamed Ibrahim

Reviewer #3: No

Reviewer #4: **Yes:** Mukhyaprana Manuru Prabhu

[NOTE: If reviewer comments were submitted as an attachment file, they will be attached to this email and accessible via the submission site. Please log into your account, locate the manuscript record, and check for the action link "View Attachments". If this link does not appear, there are no attachment files.]

While revising your submission, please upload your figure files to the Preflight Analysis and Conversion Engine (PACE) digital diagnostic tool, <https://pacev2.apexcovantage.com/>. PACE helps ensure that figures meet PLOS requirements. To use PACE, you must first register as a user. Registration is free. Then, login and navigate to the UPLOAD tab, where you will find detailed instructions on how to use the tool. If you encounter any issues or have any questions when using PACE, please email PLOS at [figures@plos.org](mailto:figures@plos.org). Please note that Supporting Information files do not need this step.
